# Spinach (*Spinacia oleracea*) has epidermal bladder cells and exhibits characteristics of a facultative halophyte

**DOI:** 10.1038/s42003-025-08936-6

**Published:** 2025-11-29

**Authors:** Clarissa Buarque Vieira, Jorge F. S. Ferreira, Devinder Sandhu, Sol Sepsenwol, Maria Betânia G. S. Freire

**Affiliations:** 1 Federal Rural University of Pernambuco (UFRPE), Dois Irmãos, Recife, Brazil; 2 U.S. Salinity Laboratory (USDA-ARS), Riverside, CA USA; 3 Department of Biology, University of Wisconsin-Stevens Point, Stevens Point, WI USA

**Keywords:** Salt, Leaf development

## Abstract

While epidermal bladder cells (EBCs) have been well documented in quinoa, their presence in spinach has not been previously reported. Spinach is traditionally classified as a glycophyte but our previous and current results strongly suggest otherwise. In this study, we compared two quinoa with two spinach cultivars grown under low (2 dS m⁻¹) and high (25 dS m⁻¹) irrigation-water salinities. Using Scanning Electron Microscopy coupled with Energy-Dispersive X-ray Spectroscopy (SEM-EDS), we demonstrated that spinach has EBCs that accumulate mostly Cl, followed by K, and to a lesser extent Na, while quinoa EBCs showed no detectable Na signal. Morphologically, quinoa EBCs have single-celled stalks while spinach EBCs have multicellular stalks; both feature globular heads approximately 150 µm in diameter. Spinach displayed higher expression of Na- and Cl-transporter genes in EBCs than in EBC-free leaf tissue. Under identical salinity levels (2-24 dS m⁻¹), both spinach and quinoa exhibited comparable reductions in biomass and plant height. Despite higher Na and Cl accumulation, spinach exhibited no visible signs of salt toxicity. The presence of EBCs and apparent tissue-level salt tolerance provide new insights into spinach anatomy and ion partitioning under salinity stress, suggesting that spinach may be more accurately classified as a facultative halophyte.

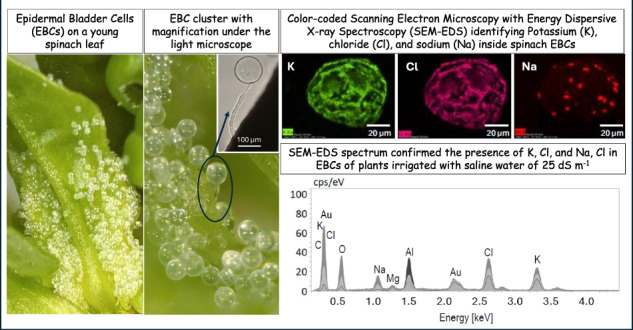

## Abstract (web summary)

Spinach has epidermal bladder cells that accumulate Cl and K, a feature not previously documented in this species. Like quinoa, spinach maintains growth under salinity, suggesting facultative halophyte status and unique ion partitioning.

## Introduction

Soil salinization and sodification are rapidly increasing in agricultural lands worldwide, particularly in arid and semiarid regions. Recent studies demonstrate that over 1 billion hectares are affected by salts in more than 100 countries and that soil salinization is estimated to expand by approximately three arable hectares per minute, although there are no directly measured global inventories of soil salinity^1–3^. This trend is driven by natural and anthropogenic factors; among the latter, industrial activities, inadequate irrigation, excessive fertilization, and brackish water use^4,5^. Also, coastal seawater intrusion replaces over-tapped groundwater, which becomes more saline and increases water/soil salinization. Currently, saline/sodic areas are being abandoned as they become unsuitable for the cultivation of most crops. Since soil and freshwater are the foundations of agriculture, most of the current staple crops are sensitive to soil salinity and degradation, threatening agricultural food and feed production, resulting in significant socioeconomic, environmental, and food-security challenges.

One alternative for the use and/or mitigation of saline soils is the cultivation of salt-tolerant species and cultivars^6^. The ability of plants to survive in saline soil largely depends on their mechanisms for circumventing salinity stress. Tolerance mechanisms include extrusion of Na^+^ and Cl^−^ from roots and shoots, sequestration of Na^+^ and Cl^−^ into vacuoles, Na^+^ and Cl^−^ exclusion from the transpiration stream, osmotic regulation, tissue tolerance to high salt accumulation, and potassium homeostasis^7,8^. Approximately 1–2% of plant species are classified as halophytes, including *Chenopodium quinoa* Willd., *Chenopodium album* L., and *Atriplex spp*. that harbor specialized leaf epidermal structures called epidermal bladder cells (EBCs)^9^. Hence, studies on the ionic and genetic mechanisms related to salt-ion compartmentalization in EBCs are deemed essential to further understand plant salt tolerance and to advance genetic modifications in glycophytes, enabling them to maintain sustainable food production in saline soils^1,10–12^.

The direct role of EBCs in salt tolerance and survival of halophytes in saline and sodic soils^13^ has been currently challenged^14^. Previously, the removal of quinoa EBCs from leaves reportedly reduced Na^+^ and Cl^−^ tolerance and compromised leaf K^+^ retention, resulting in a salt-sensitive phenotype^15^. However, subsequent attempts to replicate this finding were reported as unsuccessful^14,16^. In contrast, quinoa glandless mutants had no reduction in salt tolerance compared to glanded wild-type plants^14^. Furthermore, quinoa EBCs preferentially took up K^+^ over Na^+^; similar to the preference for K^+^ over Na^+^ in spinach under salt stress^17^. Thus, although EBCs’ role has been challenged, some studies identified higher expression of key anion (*SLAH*, *NRT*, *CLC*) and cation (*NHX1*, *HKT1*) transporter genes in EBCs compared to surrounding leaf cells in halophytes^12,18^.

Spinach (*Spinacia oleracea* L., Amaranthaceae), has traditionally been classified as a glycophyte, with a salinity-tolerance threshold of 2.0 dS m^−1^, based on soil-paste electrical conductivity (EC_e_)^19,20^. However, a study conducted at the US Salinity Laboratory in Riverside challenged this low-salinity threshold^21^. That study evaluated the salinity response of ʻRaccoonʼ spinach to salinity during three seasons and determined that as temperatures increased, salinity tolerance decreased. Raccoon' salinity threshold was equivalent to an EC_e_ = 4.25 dS m^−1^, higher than previously reported^19,20^. Later studies with ʻRaccoonʼ and ʻGazelleʼ demonstrated that these cultivars had salinity-threshold EC_e_ of 6.1 and 8.0 dS m^−1^, based on the electrical conductivities of the irrigation water (EC_iw_) of 13.0 and 17.0 dS m^−1^, respectively^17,22^. Despite accumulating 5% Na and 7% Cl in their leaves, spinach plants favored the absorption of K^+^ over Na^+^ and maintained tissue homeostasis of N, P, and K^17,22^. These results indicate that spinach can override the commonly cited competition between Na^+^ vs. K^+^ and Cl^−^ vs. NO_3_^−^, a characteristic typically associated with halophytes. Additionally, ʻRaccoonʼ and ʻGazelleʼ exhibited minimal leaf-biomass loss under an EC_iw_ of 17 dS m^−1^, even when salinity was combined with potassium fertilization at levels over 40-fold lower than required for the crop^22^. This ability to maintain potassium homeostasis under high salinity and K deficiency is a characteristic typically observed in halophytes.

A recent study evaluating 16 regionally diverse spinach cultivars identified some with even greater salt tolerance than ʻRaccoonʼ and ʻGazelleʼ^23^. The most tolerant cultivars survived a calculated EC_e_ = 10.86 dS m^−1^, over five-fold higher than the often-cited threshold (EC_e_ of 2.0 dS m^−1^)^24^. Even though spinach plants experienced a reduction in shoot biomass and accumulated macronutrient-levels of Na and Cl in their leaves when exposed to high salinity (EC_iw_ = 17 dS m^−1^)^22^, they showed no visual signs of salt damage. Furthermore, plants maintained baseline levels of N, P, and K, absorbed considerably high Na when K was deficient, but favored K over Na when K levels were restored in the irrigation water. Spinach plants also increased (ʻRaccoonʼ) or maintained (ʻGazelleʼ) shoot biomass when NaCl increased from 5 to 60 mM^17^; all characteristics commonly associated with halophytes. Although quinoa is a halophyte, its biomass may also decrease under varying levels of salinity stress, with its salt tolerance depending on the genotype or cultivar^13,25^. Spinach also exhibits varying salt tolerance across different cultivars^23^.

While specialized epidermal structures as EBCs have been investigated in halophytes^10^, EBCs have not yet been reported in spinach. Spinach was previously reported to lack EBCs^1^, although the authors did not mention the specific cultivar. Spinach classification as a glycophyte likely originates from a salt-sensitive cultivars with low-salinity threshold (EC_e_)^24^, which in turn precluded researchers from looking for EBCs in an “extremely salt-sensitive glycophyte”.

Scanning electron microscopy (SEM) coupled with energy-dispersive X-ray spectroscopy (EDS) is widely used to analyze the elemental composition of samples. SEM-EDS allows for the detection of elements, their relative concentrations, and spatial distribution at micro- to nanometer scales. It is a semi-quantitative technique with a typical limit of detection of approximately 1000 ppm for elements with higher atomic masses (e.g., Na, Cl, K) and around 100 ppm for lighter elements such as F and Be (https://www.jeolusa.com/RESOURCES/Electron-Optics/Documents-Downloads/can-i-trust-my-quantitative-eds-data?). SEM-EDS has been increasingly used to study ion accumulation in plant trichomes, including recent applications for measuring K, Cl, Ca, and Mg in tomato glandular trichomes and Na and K in quinoa EBCs^26,27^.

To our knowledge, we provide the first evidence that spinach possesses EBCs; we verify their presence across 13 cultivars and compare EBC morphology and ion accumulation in two spinach cultivars and two quinoa genotypes. Our findings demonstrate that spinach EBCs accumulate Cl and K as major ions, with a smaller but measurable accumulation of Na, with both Na and Cl levels increasing under high salinity. Conversely, quinoa EBCs had no detectable Na but accumulated more K than spinach EBCs. Furthermore, our analysis in spinach reveals distinct expression patterns of genes involved in Na, Cl, and K transport between EBCs and leaves lacking EBCs. The combination of SEM-EDS, tissue salt accumulation, and gene expression allowed us to integrate morphological, ionomic, and genetic aspects to further our insight into the mechanisms of salt partitioning in spinach.

## Results

### EBCs morphology

**Fig. 1:**
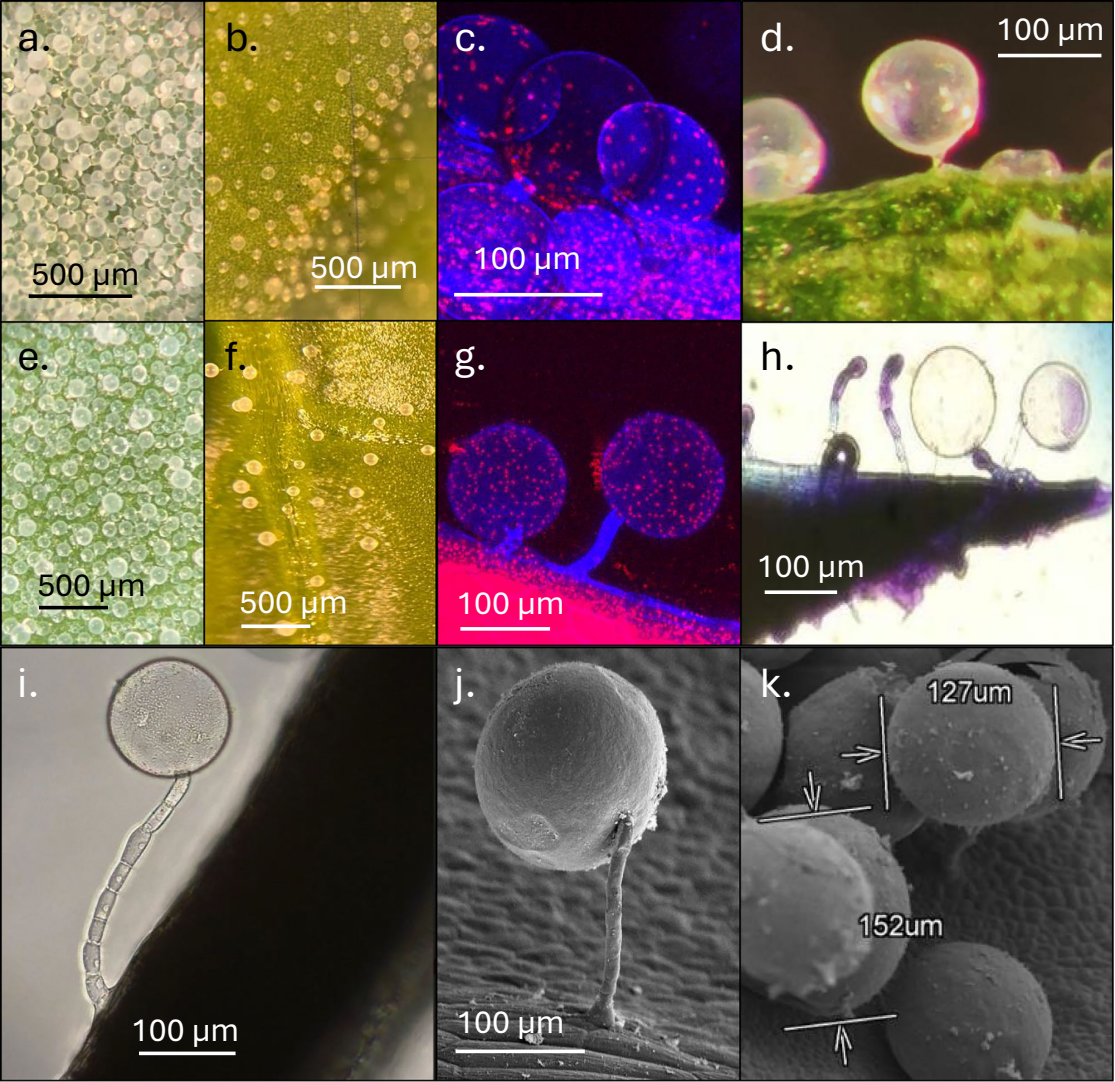
Morphology of epidermal bladder cells (EBCs) in quinoa and spinach. **a** EBCs on young quinoa leaf at low power. **b** EBCs on mature quinoa leaf at low power. **c** Confocal image of unstained quinoa EBCs, showing internal autofluorescence. **d** Quinoa EBCs at higher magnification. **e** Spinach EBCs on young leaf at low power. **f** Spinach EBCs, on mature leaf at low power. **g** Confocal image of unstained spinach EBCs, showing internal autofluorescence. **h** Spinach EBCs, showing early to late developmental stages. **i** Mature spinach EBC showing stalk cells at higher magnification. **j** Scanning electron microscope (SEM) image of FAA-fixed spinach EBC, showing multicellular stalk. **k** SEM image of spinach EBC showing bladder size variation.

**Fig. 2:**
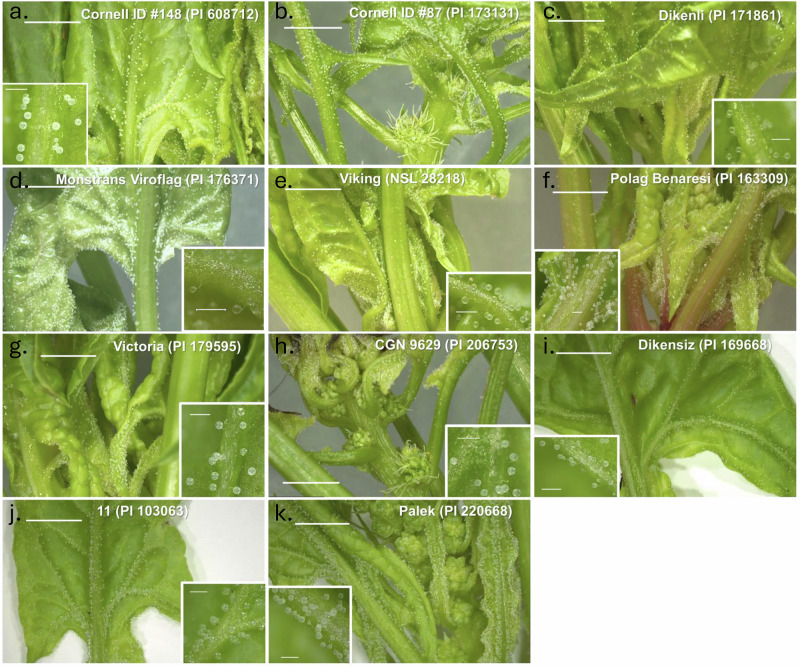
Epidermal bladder cells (EBCs) in 11 spinach varieties. EBCs are most abundant over the entire abaxial surface of very young leaves, particularly along leaf veins and margins. **a** Cornell ID #148. **b** Cornell ID #87. **c** Dikenli. **d** Monstrans Viroflag. **e** Viking. **f** Polag Benaresi. **g** Victoria. **h** CGN 9629. **i** Dikensiz. **j** 11. **k** Palek. In older leaves, EBCs were primarily concentrated along veins. Insets show consistent EBC structure across all cultivars: a multicellular stalk extending from the epidermis to a large, transparent bladder cell. Scale bar for main image = 5 mm; scale bar for inset = 0.5 mm.

According to the trichome classification of *Solanum* species^28^, the trichomes in quinoa can be characterized as EBCs. Microscopic evaluation of two spinach cultivars revealed that spinach also possesses modified trichomes like the EBCs found in quinoa (Fig. 1a–d). Spinach EBCs (Fig. 1f) are not as numerous as quinoa’s (Fig. 1b) but have the same bluish fluorescence as quinoa EBCs (Fig. 1c, g) under fluorescence microscopy, surrounded by red fluorescence of leaf epidermal cells. Quinoa EBCs feature a large globular head supported by a single stalk cell (Fig. 1c), whereas spinach EBCs consist of a large globular head with a multicellular stalk (Fig. 1h, i). The overall length of the stalk varies slightly among different EBCs (Fig. 1h–j), and the diameter of the bladder cell ranges from 125 to 155 µm (Fig. 1k). Later evaluation of 11 spinach genotypes shows that EBCs were present in all cultivars (Fig. 2). EBCs were most abundant on the abaxial surface of very young leaves, especially along the veins and margins, and remained visible on older leaves, where they were primarily concentrated along the veins (Fig. 2).

### SEM-EDS

**Fig. 3:**
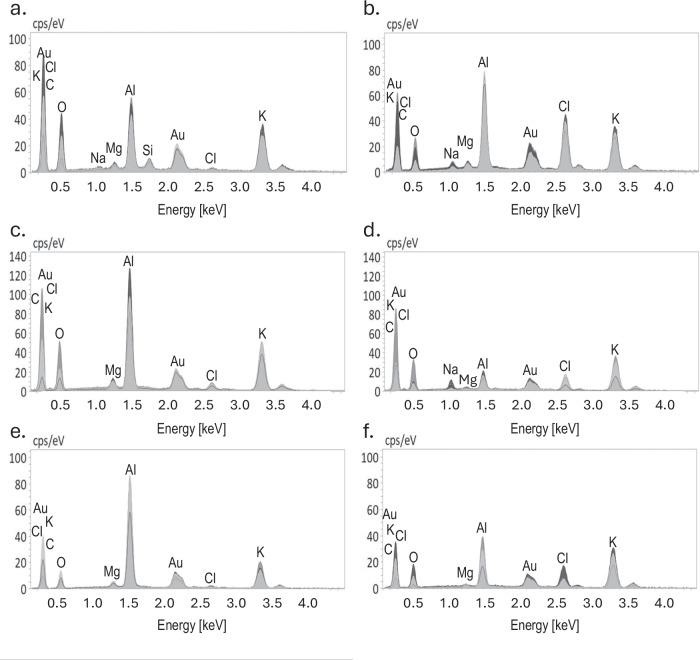
SEM-EDS spectra of spinach and quinoa EBCs under two salinity levels. **a** Spinach (ʻGazelleʼ) EBCs at EC_iw_ of 2 dS m^−1^. **b** Spinach (ʻGazelleʼ) EBCs at EC_iw_ of 25 dS m^−1^. **c** Spinach (ʻSeasideʼ) EBCs at EC_iw_ of 2 dS m^−1^. **d** Spinach (ʻSeasideʼ) EBCs at EC_iw_ of 25 dS m^−1^. **e** Quinoa (ʻCPAC09ʼ) EBCs at EC_iw_ of 2 dS m^−1^. **f** Quinoa EBCs at EC_iw_ of 25 dS m^−1^. Shaded peaks represent the relative intensity (counts per second per electron volt, cps/eV) for each detected element, and labels indicate the corresponding elemental identity.

**Fig. 4:**
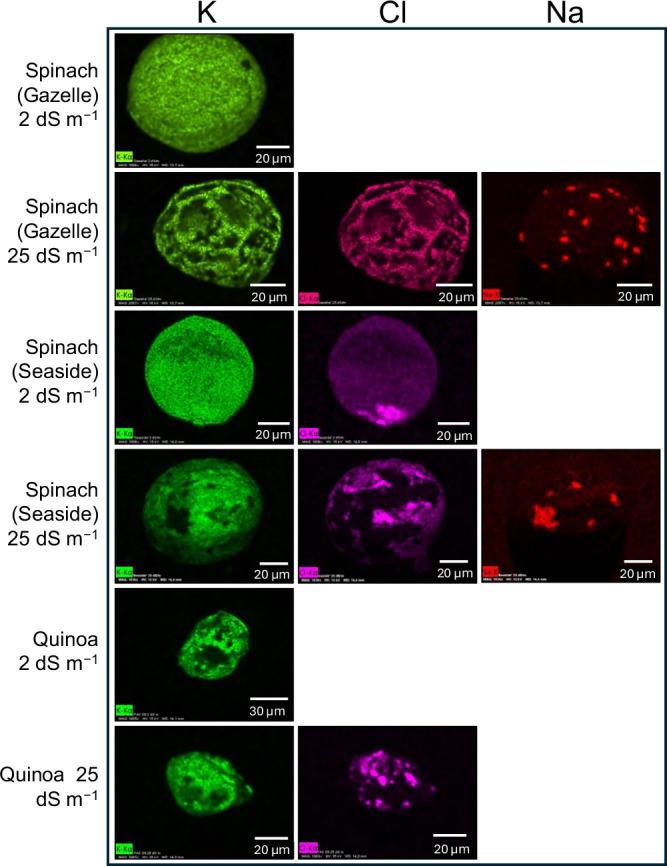
SEM-EDS ion distribution maps of K, Cl, and Na in spinach and quinoa EBCs grown under two salinity levels. Columns show K, Cl, and Na maps; rows (top to bottom) are: ʻGazelleʼ (2 dS m^−^^1^), ʻGazelleʼ (25 dS m^−^^1^), ʻSeasideʼ (2 dS m^−^^1^), ʻSeasideʼ (25 dS m^−^^1^), quinoa (2 dS m^−^^1^), and quinoa (25 dS m^−^^1^). Intensities are displayed as false-color heatmaps, where brighter tones indicate higher relative EDS counts.

SEM-EDS analysis revealed that both spinach and quinoa allocate salts inside EBCs (Figs. 3 and 4). Spinach EBCs contained K, Cl, and Na (Figs. 3a–d and 4). Under control (low-salinity) conditions of EC_iw_ = 2 dS m^−1^ the EBCs of spinach plants exhibited higher concentrations of K compared to Na and Cl (Fig. 3a, c). However, when spinach plants were submitted to EC_iw_ of 25 dS m^−1^ (Fig. 3b, d) the concentrations of Na and Cl increased, mainly for ʻGazelleʼ (Fig. 3b). Like spinach, the presence of K and Cl was observed in quinoa EBCs (Fig. 3e, f). Contrary to spinach, there was no detectable Na peak in quinoa EBCs, regardless of salinity level (Fig. 3e, f). When low-salinity water was applied to quinoa, the content of Cl was insignificant (Fig. 3e). However, the concentrations of Cl and K significantly increased with water of EC_iw_ of 25 dS m^−1^ (Fig. 3f). Specifically, EBCs signal for K increased from 20 to 30 counts per second per electron-volt (cps/eV) when salinity increased from 2 to 25 dS m^−1^ (Fig. 3e, f). All SEM-EDS spectra show large peaks for aluminum (Al) and gold (Au), generated by the aluminum stub supporting the glands and gold used to coat the dry glands, respectively (Fig. 3).

**Fig. 5:**
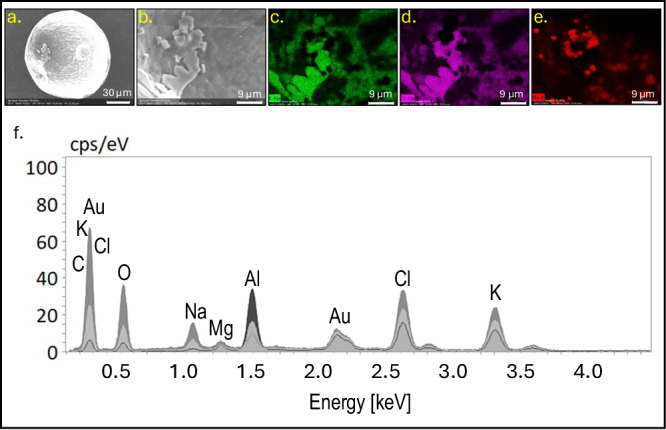
SEM-EDS ion distribution maps and spectra of air-dried spinach EBCs. **a** Air-dried spinach EBC. **b** Salt crust inside air-dried spinach EBC. **c** K distribution inside air-dried spinach EBC. **d** Cl distribution inside air-dried spinach EBC. **e** Na distribution inside air-dried spinach EBC. **f** SEM-EDS spectra of air-dried spinach EBCs. Shaded peaks represent relative intensity (counts per second per electron volt, cps/eV) for each detected element, and labels indicate the corresponding elemental identity.

According to the SEM-EDS distribution maps, Cl and K were colocalized in spinach EBCs, while Na displayed a distinct localization pattern (Fig. 4). Similarly, in quinoa, Cl and K were also colocalized (Fig. 4). After the leaves were washed with distilled, deionized water and EBCs were removed and air-dried (Fig. 5a), a salt crust was revealed on the inner surface of the spinach EBCs (Fig. 5b). The mapping shows K and Cl distribution signatures in a similar pattern (Fig. 5c, d) as the salt crust (Fig. 5b), while Na was segregated from K and Cl (Fig. 5e). Disregarding Au and Al, as explained earlier, the SEM-EDS graph after 1.0 keV exhibits the intensity of the mineral salts Na, Mg, Cl, and K that were all present in the crust (Fig. 5f).

Semi-quantitative data from SEM-EDS confirmed that both EBCs of spinach and quinoa had K and Cl, but there was no detectable signal for Na in quinoa EBCs (Table 1). For quinoa plants irrigated with control water of 2 dS m^−1^, ʻCPAC09ʼ had significantly more K than ʻCPAC11ʼ, the same was observed at 25 dS m^−1^, although not significantly. For spinach plants, there was no difference for K concentrations between genotypes or salinity treatments. In general, quinoa EBCs had significantly more K than spinach plants when irrigated with high-salinity water (Table 1).

**Table 1:** Concentration of K, Cl, and Na in EBCs of two cultivars of quinoa and spinach estimated by Scanning Electron Microscopy (SEM) combined with Dispersive X-ray Spectrometry (EDS)

EC_iw_^*^	Quinoa cultivar		Spinach cultivar	
	CPAC09	CPAC11	Gazelle	Seaside
K (g kg^−1^)				
2	257.83 ± 14.35Ba	174.2 ± 39.59Bb	112.13 ± 38.15Ab	181.03 ± 2.15Aab
25	317.00 ± 67.40Aa	240.4 ± 21.10Aa	118.37 ± 16.95Ab	160.23 ± 15.83Ab
Mean	287.42a	207.3b	115.25c	170.63b
Cl (g kg^−1^)				
2	2.33 ± 4.04Ba	11.77 ± 14.15Ba	0.00 ± 0.00Ba	29.53 ± 16.65Ba
25	40.30 ± 0.16.90Ab	82.00 ± 23.20Aa	94.40 ± 6.79Aa	69.90 ± 5.10Aab
Mean	21.31b	46.88a	47.20a	49.71a
Na (g kg^−1^)				
2	0.00 ± 0.00Aa	0.00 ± 0.00Aa	5.00 ± 5.00Ba	0.00 ± 0.00Ba
25	0.00 ± 0.00Ab	0.00 ± 0.00Ab	15.67 ± 8.58Aa	10.33 ± 4.18Aa
Mean	0.0b	0.0b	10.33a	5.17ab

The values in g/kg of each element were calculated from the normalized element mass concentration (in %) provided by each element intensity in a spectrum signal generated by its energy obtained by SEM-EDS at 15 keV. Mean values come from three different values originated for each epidermal bladder cell (EBC) and from three different EBCs by SEM-EDS. Values are the means ± SD (standard deviation) of three replicates. Uppercase letters compare values in each column for each mineral and lowercase letters compare values in each row. Means compared using Tukey test (*p *≤ 0.5).

^*^EC_iw_ Electrical conductivity of the irrigation water in deciSiemens per meter (dS m^−1^).

Under low-salinity irrigation, Cl concentrations in EBCs were similarly low across both quinoa and spinach genotypes, with no cultivar differences (Table 1). At high salinity, Cl levels increased significantly in all genotypes. However, while quinoa showed a cultivar-specific difference, spinach did not. Quinoa ʻCPAC11ʼ accumulated two-fold more Cl than ʻCPAC09ʼ. Overall, Cl concentrations were significantly higher under high-salinity conditions in both quinoa and spinach cultivars (Table 1).

No detectable Na was observed in quinoa EBCs under either low- or high-salinity conditions, while Na levels in spinach EBCs were significantly higher under high-salinity irrigation compared to low salinity (Table 1). These semi-quantitative findings by SEM-EDS are consistent with the SEM-EDS peak intensity data presented in Fig. 3.

### Comparison of spinach and quinoa under different salinity treatments

**Fig. 6:**
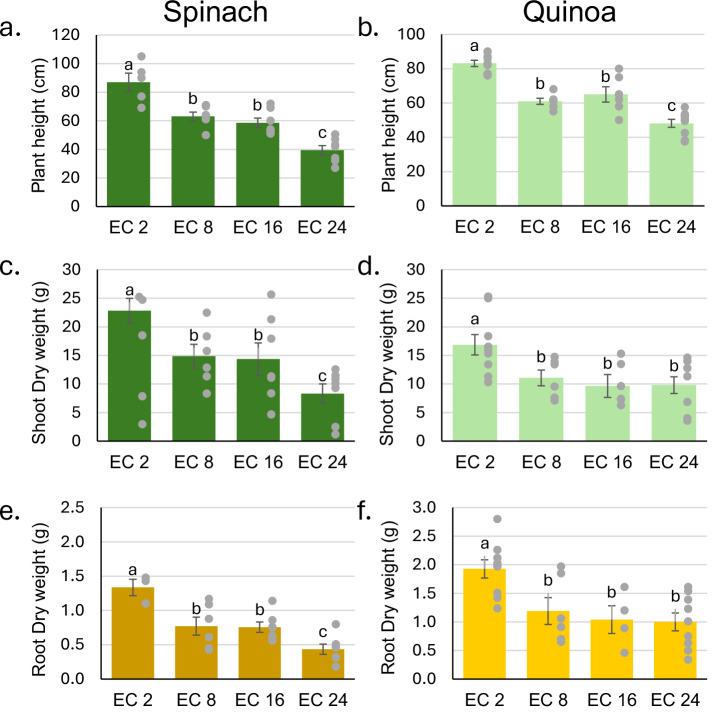
Performance of spinach (ʻCGN 9629ʼ) and quinoa (ʻCPAC11ʼ) under four salinity treatments. **a** Spinach plant height. **b** Quinoa plant height. **c** Spinach shoot dry weight. **d** Quinoa shoot dry weight. **e** Spinach root dry weight. **f** Quinoa root dry weight. Means represented by the same letters are not significantly different according to Tukey’s HSD (0.05), *n* = 9 biologically independent samples. Error bars represent standard errors.

To compare salinity tolerance between spinach and quinoa, four levels of irrigation-water electrical conductivity (EC_iw_) were tested: 2 (control), 8, 16, and 24 dS m^−^^1^. Both spinach ʻCGN 9629ʼ and quinoa ʻCPAC11ʼ had significant reductions in plant height at 8 dS m^−^^1^ (Fig. 6a, b), while neither species differed in height between 8 and 16 dS m^−^^1^. Then, both species reduced height significantly from 16 to 24 dS m^−^^1^. Spinach showed height reductions of 28%, 33%, and 55% at 8 dS m^−^^1^, 16 dS m^−^^1^, and 24 dS m^−^^1^, respectively, while quinoa showed reductions of 27%, 22%, and 42% at the same salinity levels. Thus, reductions in plant height were similar and significant for both species when water salinity increased from control to 24 dS m^−^^1^ (Fig. 6a, b).

Shoot dry weight followed a similar trend. Both species showed significant reduction at 8 dS m^−^^1^, with no major differences between 8 and 16 dS m^−^^1^ for spinach, followed by a significant drop for spinach, but not quinoa, at 24 dS m^−1^ (Fig. 6c, d). At the highest water salinity, spinach shoot biomass was reduced by 64%, compared to 42% in quinoa.

Root dry weight mirrored the shoot dry weight pattern (Fig. 6e, f). At 24 dS m^−1^, spinach roots showed a 67% reduction, while quinoa roots were reduced by 48%. The responses of plant height, shoot, and root dry weight were very similar for spinach and quinoa indicating that quinoa was only a bit more salt tolerant than spinach and depending on the cultivar, both species can suffer measurable salt stress.

### Tissue mineral accumulation

Leaves of spinach plants accumulated significantly more Na and Cl than quinoa (Table 2). Spinach accumulated 12-fold more Na than quinoa (12.3 vs. 1.0 g kg^−1^) at an EC_iw_ of 2 dS m^−1^ (low salinity), and nearly 4.5-fold higher than quinoa leaves (78.7 vs. 17.5 g kg^−1^) at the EC_iw_ of 25 dS m^−1^ (high salinity). While Cl accumulation in spinach and quinoa leaves was similar at low salinity (20.4 vs. 16.5 g kg^−1^), Cl concentration in spinach leaves was almost twice as high as that of quinoa leaves (156.2 vs. 80.9 g kg^−1^) at high salinity (Table 2). Quinoa accumulated 70.3 g kg^−1^ of K when irrigated with water of low salinity (similar to 59.5 g kg^−1^ accumulated in spinach leaves). However, quinoa leaves accumulated 94.3 g kg^−1^ of K, compared to 31.4 g kg^−1^ in spinach, when plants were irrigated with high-salinity water. Thus, quinoa increased leaf accumulation of K by approximately 34% under salinity stress, while spinach leaf accumulation of K under salinity stress was reduced by approximately 47% (Table 2).

**Table 2:** K, Na, and Cl concentration in spinach (ʻGazelleʼ) and quinoa (ʻCPAC09ʼ) leaves under EC_iw_ of 2 and 25 dS m^−1^

EC_iw_ (dS m^−1^)	Ion Concentration (g kg^−1^ dry weight)					
	K		Na		Cl	
	Spinach	Quinoa	Spinach	Quinoa	Spinach	Quinoa
2	59.4Ab	70.3Ba	12.3Ba	1.0Bb	20.4Ba	16.5Ba
25	31.4Bb	94.3Aa	78.7Aa	19.1Ab	156.2Aa	80.9Ab
ANOVA	F		F		F	
Species	189.25^*^		97.46^*^		32.98^*^	
EC_iw_	0.54^NS^		127.40^*^		211.16^*^	
Species X EC_iw_	93.88^*^		46.23^*^		26.87^*^	
CV (%)	8.4		26.82		20.12	

Uppercase letters compare means in each column and lowercase letters compare means in each row for each mineral—means compared using the Tukey test at 5% probability.

^*^ and ^NS^ are equal to 0.1% and no significant, respectively.

Although salt mineral accumulation occurred in roots under low salinity, Na and Cl levels did not differ between species, whereas spinach tended to accumulate more K in roots compared to quinoa (Table 3). At 25 dS m^−1^, the accumulation of K, Na, and Cl was significantly higher in spinach than quinoa. Both spinach and quinoa roots maintained their K concentrations regardless of irrigation-water salinity (Table 3).

**Table 3:** K, Na, and Cl concentration in spinach (ʻGazelleʼ) and quinoa (ʻCPAC09ʼ) roots under EC_iw_ of 2 and 25 dS m^−1^

EC_iw_ (dS m^−1^)	Ion Concentration (g kg^−1^ dry weight)					
	K		Na		Cl	
	Spinach	Quinoa	Spinach	Quinoa	Spinach	Quinoa
2	42.1	19.8	4.2Ba	6.5Ba	8.15Ba	6.6Ba
25	44.6	22.5	18.9Aa	15.7Ab	41.6Aa	23.0Ab
ANOVA	F		F		F	
Species	132.19^***^		0.74^NS^		39.97^***^	
EC_iw_	1.77^NS^		150.36^***^		266.00^***^	
Species X EC_iw_	0.001^NS^		8.05^*^		28.30^***^	
CV (%)	11.93		17.12		16.12	

Uppercase letters compare means in each column and lowercase letters compare means in each row for each mineral—means compared using the Tukey test at 5% probability.

^***^, ^*^ and ^NS^ are equal to 0.1%, 5% of probability and no significant, respectively.

### Expression analysis

Gene expression analysis was conducted on two distinct organs of spinach plants subjected to high salinity conditions. DNA was extracted from EBCs excised from leaves and from the residual leaf tissue devoid of EBCs. This study utilized two spinach varieties, ʻGazelleʼ and ʻSeasideʼ, to assess the impact of high salinity on gene expression in these specific plant tissues. Seventeen genes associated with salinity tolerance in spinach were selected based on their roles and mechanisms related to salt-stress responses (Supplementary Table 1). The gene selection encompassed genes that regulate Na levels, including *SOS1*, *SOS2*, *SOS3*, *NHX1*, *NHX2*, and *HKT1*; potassium homeostasis, such as *AKT1*, and chloride regulation, through the expression of *CLCc*, *CLCg*, *SLAH1*, *NPF2.4*, *ALMT12*, and *CCC*. Additionally, four genes (*Spo09736*, *Spo11258*, *Spo15968*, and *Spo19814)* previously identified as salt-responsive in RNA-seq study^29^, were also included.

**Fig. 7:**
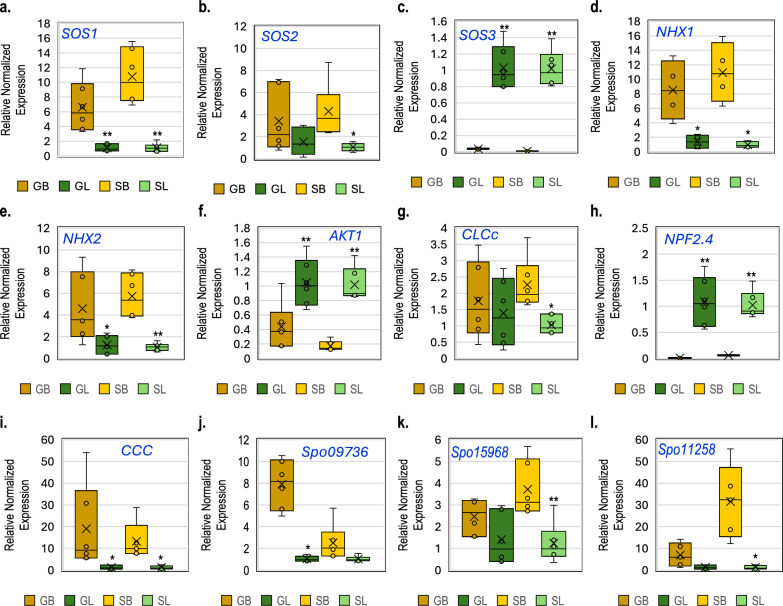
Expression of various genes involved in salt tolerance in EBCs (B) and leaves devoid of EBCs (L) of spinach plants ʻGazelleʼ and ʻSeasideʼ irrigated with saline waters of electrical conductivity of 25 dS m^−1^. **a***SOS1*. **b** *SOS2*. **c** *SOS3*. **d** *NHX1*. **e** *NHX2*. **f** *AKT1*. **g** *CLCc*. **h** *NPF2.4*. **i** *CCC*. **j** *Spo09736*. **k** *Spo15968*. **l** *Spo11709*. For boxplots, horizontal line indicates the median, boxes the interquartile range, “x” the mean, whiskers 1.5 × IQR, and points individual biological replicates. Asterisks denote significance for EBC vs EBC-free leaf comparisons (two-tailed t-test): *P* ≤ 0.05 (*), *P* ≤ 0.01 (**). Error bars represent standard errors. *n* = 3 biological replicates.

In the analysis of genes regulating Na levels, *SOS1* was significantly upregulated (*P* > 0.01) in EBCs of both varieties, while *SOS2* was upregulated only in EBCs of ʻSeasideʼ (Fig. 7a, b). In contrast, *SOS3* exhibited a notable and highly significant (*P* > 0.01) downregulation in EBCs compared to leaves lacking EBCs across both spinach cultivars (Fig. 7c). Meanwhile, both *NHX1* and *NHX2* showed significantly higher expression in EBCs relative to the surrounding leaf tissue (Fig. 7d, e). The K^+^ channel gene, *AKT1* was significantly downregulated in EBCs of both varieties compared to the leaves without EBCs (Fig. 7f). Interestingly, *HKT1*, a Na transporter, was not differentially expressed in EBCs and EBCs-less leaves (Supplementary Fig. 1a).

For genes responsible for Cl regulation, *CLCc*, and *CCC*, demonstrated significant upregulation in EBCs compared to EBC-less leaves of at least one cultivar (Fig. 7g, i), but *NPF2.4* was significantly downregulated in EBCs of both varieties (Fig. 7h). Meanwhile, *CLCg*, *SLAH1*, and *ALMT12* were not differentially expressed in EBCs and EBCs-less leaves (Supplementary Fig. 1b–d).

Among the four salt-responsive genes identified via RNA-seq, *Spo09736*, *Spo15968*, and *Spo11258* were upregulated in EBCs (Fig. 7j–l), while *Spo19814* showed no difference in expression between the two tissue types (Supplementary Fig. 1e). This comprehensive gene selection provided a broad, yet specific, insight into the genetic basis of salt accumulation in spinach EBCs.

## Discussion

While EBCs have been documented in other Amaranthaceae members like *C. quinoa* and *Atriplex* sp., we found no reports of EBCs in spinach in the literature. One study specifically noted that spinach lacks these specialized structures, but gave no cultivar or source of reference^1^. We demonstrated that spinach leaves possess glandular trichomes with features similar to quinoa EBCs. In this study, we examined a total of 13 spinach cultivars and found EBCs present in all of them (Figs. 1 and 2). The consistent presence of EBCs across diverse cultivars suggests that these structures are a highly conserved anatomical trait in spinach. This finding expands our previous knowledge of spinach’s physiological and ionomic adaptations to high salinity^17,22,23,30^, highlighting the presence of EBCs, previously unrecognized in this species.

The structural similarities between quinoa and spinach glandular trichomes suggest that those in spinach may also function as EBCs, potentially playing similar roles as quinoa. The development of spinach EBCs may evolve from the basal cells of the filamentous stem structure, as other glandular trichomes, where the terminal cell later expands into a bladder (Fig. 1f). These EBCs may produce a range of secondary metabolites that represent an advantage to the survival of the species by sequestering chemical defense compounds against insects, as recently reported for quinoa EBCs^31^. A phylogenetic analysis of these species estimated that *C. quinoa* and *S. oleracea* separated about 16 million years ago^12^, so they may retain function similarities in their EBCs.

Although quinoa EBCs have a similar morphology to those of spinach (Fig. 1a, b, c, e, f, g), they differ significantly in stalk structure: quinoa EBCs feature single-celled stalks (Fig. 1d) while spinach EBCs have multicellular stalks (Fig. 1g–j). The density of quinoa EBCs on both young and mature leaves was higher (Fig. 1a, b) compared to spinach leaves (Fig. 1e, f). Internally, EBCs of both species (Fig. 1c, g) exhibit a blue autofluorescence that indicates the presence of phenolic compounds^32^, while the red autofluorescence inside EBCs and epidermal leaf cells indicates the presence of chloroplasts (Fig. 1c, g). Randomly distributed chloroplasts, without grana, were reported from quinoa EBCs^18^. Chloroplasts have been also reported in the 10-celled glandular trichomes from leaves and flowers of *Artemisia annua*^33,34^. This suggests that chloroplasts may provide energy for the biosynthesis of protective flavonoids and other bioactive metabolites in spinach EBCs and to operate ionic pumps. These pumps are responsible for transporting H^+^, Na^+^, Cl^−^, and K^+^ into the EBCs and ensure that excessive salt ions are sequestered from the cytoplasm^35^. Although spinach EBCs take up Cl, K, and to some degree Na, further research is needed to prove that spinach EBC have ion transport pumps powered by chloroplasts.

Our SEM-EDS findings clearly demonstrate that the EBCs of spinach leaves contain salt ions, like the EBCs of quinoa. The accumulation of K and Cl was evident in spinach EBCs, while Na accumulated at much lower levels. Notably, Na levels increased modestly under high salinity, particularly in the EBCs of ʻGazelleʼ (Table 1; Figs. 3 and 4). Quinoa EBCs showed an increase in the peak intensity for K when salinity increased from low (2.0 dS m^−1^) to high (25 dS m^−1^). This is consistent with previous reports^14,36^ and with our current results (Tables 2 and 3) that quinoa significantly increased K accumulation in leaves, but not roots, when water-salinity increased from 2 to 25 dS m^−1^. Under low-salinity irrigation water, our SEM-EDS results revealed that both spinach and quinoa EBCs mainly accumulated K (Fig. 3 and Table 1), agreeing with our results of K being the main element accumulating in leaves of both spinach and quinoa plants irrigated with low-salinity water (Table 2). These observations suggest that K play a crucial role in the ability of both species to maintain K homeostasis during salinity stress, as previously reported when spinach plants were provided 20 and 40 times less K through saline irrigation waters^17,22^. In these studies, spinach plants have been shown to favor K over Na when given enough K and to keep K leaf homeostasis (3–4% K) under moderate to high salinity (13 to 17 dS m^−1^), even when both Na and Cl had accumulated in leaf tissues to levels of 5% Na and 7% Cl^17,22^.

Our SEM-EDS data provides evidences that spinach EBCs sequester Cl and K, and to some extent Na, while quinoa EBCs primarily sequester K and Cl, with no detectable Na even at high-salinity (Fig. 3), consistent with previous report that Na accumulates mainly in the stem of quinoa^27^. This discovery abolishes previous assumptions on spinach having no EBCs and opens new avenues for further research into potential roles of EBCs, such as K homeostasis in spinach and quinoa.

Spinach leaves accumulated higher concentrations of Na than quinoa under both low and high salinity conditions, while K accumulation was significantly higher in quinoa than in spinach under both salinities (Table 2). The low tissue accumulation of Na and Cl with a concomitant increase in K (from 7 to 9.4%) are recognized as effective mechanisms of salinity tolerance in halophytes and quinoa has a higher control of Na and accumulation of K than spinach. However, the fact that spinach leaves accumulate more Na (7.8%) and Cl (11.8%) than quinoa (1.7% Na and 8.1% Cl), while maintain sufficient K levels for growth despite a reduction from 5.9 to 3.1% (Table 2), suggests that spinach tolerates salinity primarily through high tissue tolerance to both Na and Cl. This is further supported by the lack of visual salt-toxicity symptoms and challenges its current classification as a glycophyte. Root accumulation of Na and Cl seemed to be under some level of control, but concentrations were significantly higher in spinach than in quinoa, and both species accumulated similar concentrations of K in roots (Table 3).

The higher control of Na and Cl transport to leaves, and significantly higher accumulation of K in quinoa under high salinity could grant quinoa an upper hand over spinach for salt tolerance. However, after the significant loss in shoot biomass of both species to salinity stress (Fig. 6), it seems that the high tissue tolerance of spinach to NaCl, and modest K homeostasis, may compensate for its lack of control in Na and Cl absorption under salinity stress. Although these responses may vary among cultivars, quinoa’s regulated absorption of Na and Cl, spinach’s high tissue tolerance to these ions, and the maintenance of K homeostasis reflect key physiological traits commonly observed in salt-tolerant species.

In a previous study, spinach plants irrigated with EC_iw_ = 9.8 dS m^−1^ accumulated 2.8% Na and 4.5% Cl^30^. Similarly, quinoa plants grown under an EC_iw_ of 25 dS m^−1^ were found to contain 3.8% Na and 9.1% Cl in shoots^37^. Additionally, Na and Cl concentrations similar to those reported for spinach in this work were observed in *Atriplex halimus* subsp. *Schweinfurthii* shoots^38^. The authors concluded that after the application of solutions of 400 mM NaCl (approximately 40 dS m^−1^), and additional treatments with CaCl_2_, *Atriplex* accumulated 7.2% of Na and 11.21% of Cl in shoots, close to the concentration reported for ʻGazelleʼ spinach irrigated with high-salinity water (Table 2). Thus, the similar accumulation of Na and Cl between spinach and Atriplex suggests that spinach is more related to a facultative halophyte than to a glycophyte.

In general, the growth and salt tolerance of glycophytic plants depend on various factors, including plant genotype and age, evapotranspiration, compartmentation of Na^+^, K^+^, and Cl^−^, synthesis of compatible solutes, and leaching fraction applied. These plants typically grow best in the absence of salt, but can tolerate NaCl concentrations in the soil solution ranging from 50 to 250 mmol L^−1^ (equivalent to 2.9–14.6 g kg^−1^ or 0.3–1.5%)^9^. On the other hand, halophytic plants can accumulate 500 to 1000 mmol L^−1^ NaCl (equivalent to 29.2–58.4 g kg^−1^ or 3.0–5.8%), and complete their life cycle under these high-salt concentrations^9,39^. They can survive soil salt concentrations around 200 mM NaCl (ca. 20 dS m^−1^), concentrations that would be lethal for 99% of other species^9^. Some halophytes exhibit enhanced growth under low salinity conditions compared to non-saline controls.

In a study involving ʻRaccoonʼ and ʻGazelleʼ spinach plants submitted to varying NaCl concentrations and K doses, both spinach cultivars accumulated approximately 5.6% Na and 7.5% Cl (ʻRaccoonʼ) and over 5.6% Na and over 8.0% Cl (ʻGazelleʼ) under 160 meq L^−1^ NaCl (17 dS m^−1^), regardless of K doses^22^. Interestingly, shoot biomass for both cultivars increased (although not always significantly) with NaCl doses of 30 and 60 meq L^−1^ at the two lowest doses of K (0.07 and 0.15 meq L^−1^), and K remained stable (≥3.5%) for both cultivars when K was deficient (0.07 to 0.3 meq L^−1^). When K was provided at 3 meq L^−1^, leaf K levels increased to 4.5–6.0% for ʻRaccoonʼ and 4.5–7.5% for ʻGazelleʼ^22^. At this K dose, shoot biomass changes were minimal. The salinity tolerance of these spinach cultivars seemed to be linked to their tissue salt tolerance, the ability of using Na for growth under K deficiency (osmotic adjustment), and the ability to maintain K, N, and P homeostasis in shoots and roots, even at high tissue concentrations of Na and Cl and low K levels. Notably, the plants also maintained N and P homeostasis despite significant increases in tissue Cl at each salinity level^22^.

Halophyte plants have developed mechanisms to selectively uptake K^+^ under considerable competition with Na^+^^40^. Interestingly, spinach favors K over Na while maintaining a basic level of K, even when K was deficient in irrigation water and growing medium^17^, and showed a robust ion homeostasis mechanism for N, P, and K^17,22^, further aligning it with a halophytic behavior. A recent study demonstrated substantial variation in salinity tolerance among 16 spinach cultivars, with several performing better than ʻGazelleʼ^23^. When exposed to an EC_iw_ of 23 dS m^−^^1^, ʻGazelleʼ showed a 67% reduction in leaf biomass, whereas ʻDikenliʼ exhibited only a 25% reduction, the most salt-tolerant among the 16 cultivars tested^23^, highlighting substantial cultivar variation in salt tolerance.

In addition to documenting the morphological and ionomic characteristics of spinach, this study has enhanced our understanding of gene expression under salinity stress in spinach by comparing gene expression between EBCs and EBC-less leaf tissues under high salinity conditions. It highlights how specific genes involved in the transport of ions may play roles in the movement of ions in and out of EBCs. Key findings include the upregulation of *SOS1* in EBCs of both spinach cultivars (Fig. 7a). SOS1, a plasma membrane Na^+^/H^+^ antiporter, is part of the Salt Overly Sensitive (SOS) pathway, which plays a central role in maintaining sodium homeostasis under salt stress^41^. The pathway is typically initiated when salt stress induces cytosolic Ca^2^^+^ elevation, which is sensed by the calcium-binding protein SOS3. SOS3, in turn, activates the serine/threonine kinase SOS2, forming a complex that phosphorylates and activates SOS1 to export Na^+^ from the cytoplasm^42^. Beyond its well-known role in expelling sodium from roots into the soil, our study highlights SOS1’s critical function in facilitating sodium movement between leaf tissues and EBCs, thereby helping to maintain ion homeostasis in the leaves.

Interestingly, in contrast to *SOS1*, *SOS3* was significantly downregulated in EBCs of both cultivars (Fig. 7c), and *SOS2* was upregulated only in ʻSeasideʼ EBCs (Fig. 7b). This unexpected pattern suggests that the regulation of *SOS1* in EBCs may be partly independent of the classical SOS3–SOS2 signaling module, at least under the conditions tested. These results may reflect tissue-specific modulation of the SOS pathway, possibly tailored to the physiological role of EBCs.

The significant upregulation of *NHX1* and *NHX2* in the EBCs of both spinach cultivars indicates that vacuolar sequestration may be a key function of these specialized cells under salt stress (Fig. 7d, e). NHX1 and NHX2 proteins are key in sequestering excess sodium into vacuoles, playing a crucial role in managing sodium toxicity within plant cells^43^. Both ʻSeasideʼ and ʻGazelleʼ exhibit strong *NHX1* and *NHX2* upregulation in EBCs, indicating a conserved response among genotypes for Na^+^ compartmentalization in these cells.

Potassium is a key co-factor of several enzymes needed for cell function. The differential expression of *AKT1*, involved in K^+^ homeostasis^44^, further emphasizes the complex responses of spinach to salinity stress, potentially balancing ion homeostasis in EBCs to maintain cellular functions (Fig. 7f). Although K in spinach leaf tissues was reduced from 60 to 30 g kg^−1^ from low- to high-salinity water, 30 g kg^−1^ was enough for spinach plants to grow without showing K deficiency^17^. These authors also reported that spinach plants refused Na when K levels were increased from 0.25 to 5.0 meq L^−1^, although with a significantly reduced biomass under the salinity stress of 13 dS m^−1^.

The marked increase in expression levels of chloride regulation genes, including *CLCc* and *CCC*, exclusively in the EBCs of the ʻSeasideʼ spinach suggests a genotype-specific strategy for managing Cl levels under salinity stress (Fig. 7g, i). These genotype-specific differences were reported previously in spinach when evaluating 16 spinach genotypes from diverse geographic regions^23^.

High concentrations of Cl, like Na, pose a threat to plant health, necessitating efficient regulation mechanisms. NPF2.4, specifically, plays a pivotal role in facilitating the transport of chloride ions from the roots to the xylem^45^, indicating its crucial function in chloride movement within the plant. The consistent downregulation of *NPF2.4* in EBCs, as opposed to leaves without EBCs, across both studied genotypes, underscores its significant contribution to chloride homeostasis within EBCs. This pattern of gene expression not only reinforces the critical role of these genes in Cl^−^ homeostasis but also suggests a targeted approach by the plant to mitigate the toxic effects of high Cl levels through compartmentalization, thereby maintaining overall plant health and function in saline environments.

The genes *Spo09736*, *Spo11258*, and *Spo15968*, originally identified in RNA-seq studies under salinity stress, were upregulated in spinach EBCs in at least one cultivar (Fig. 7j–l), suggesting that these genes may play key roles in either directly supporting ion homeostasis in EBCs or by regulating broader stress-response pathways. *Spo09736* codes for expansin-like B1 (EXLB1), a protein that mitigates stress by loosening the cell wall^46^. This action enhances the cell wall’s elasticity, facilitating improved uptake of nutrients and water. *Spo11258* encodes for an E3 ubiquitin ligase, which is known to modulate stress tolerance via the abscisic acid signaling pathway^47^. *Spo15968*, encoding the S-type slow anion channel-associated homolog 2-like (SLAH2-like) protein, plays a role in nitrate transport essential for maintaining ion balance^48^. Further research is imperative to delineate the roles of EXLB1, E3 ubiquitin ligase, and SLAH2-like protein in facilitating ion homeostasis in spinach EBCs under salinity stress.

Our SEM-EDS and gene expression results are consistent with the fact that the EBCs of both spinach cultivars accumulated Cl and K, and Na to some degree (ʻGazelleʼ), and exhibited differential expressions of several ion transport-related genes compared to leaf tissue lacking EBCs. EBCs are a morphological characteristic observed and described in halophytic plants of the same botanical family (Amaranthaceae), as quinoa and *Atriplex* spp. The comparison of spinach and quinoa under different salinity treatments showed that spinach and quinoa followed a similar pattern of growth reduction in response to increasing salinity (Fig. 6). While quinoa maintained slightly higher shoot and root biomass than spinach, the overall trend of response was comparable between the two species. Given that quinoa is already classified as a facultative halophyte based on its ability to tolerate and complete its life cycle under high salinity, the similar characteristics observed in spinach strongly support its reclassification as a facultative halophyte as well. Some similarities between spinach and quinoa are explained genetically by the fact that they both belong to the family Chenopodiaceae, subfamily Amaranthaceae, and share the same common ancestor^12^. Also, our previous research with different spinach cultivars clearly showed that spinach can accumulate both Na and Cl in leaf tissues at levels higher than those of macronutrients like N, which usually accumulates to levels of 4.0% in spinach^17,22^.

Although the role of EBCs as reservoirs for excess salts and salt tolerance in quinoa has been recently contested, these structures may serve other functions in salinity response, such as maintaining K^+^ homeostasis, which remains to be further investigated. Recent evidence attest for the role of EBCs in salinity tolerance of *Atriplex canescens* by regulation of ion homeostasis and water balance^49^. However, contrasting accounts of EBCs’ roles by different authors in *C. quinoa* and in different species, such as *Atriplex* spp. indicate that further studies are needed to better understand the role of these specialized glandular trichomes in halophytes. The existence of specialized structures such as EBCs in all 13 genotypes of spinach evaluated in this study may represent an evolutionary advantage maintained through millions of years since spinach and quinoa separated phylogenetically and that may enable spinach and quinoa to cope with biotic and abiotic stresses.

In conclusion, this work provides the first description of EBCs in spinach leaves and evidence of the accumulation and partitioning of mostly Cl and K, with Na only increasing in EBCs of ʻGazelleʼ. The evidence is supported by SEM-EDS, tissue accumulation of Na, K, and Cl into leaf tissues of spinach, and gene expression analysis in both isolated EBCs and EBC-free leaf tissues from plants irrigated with high-salinity water. Our current data establishes that spinach EBCs store mostly K and Cl, particularly under high salinity, like the EBC of other halophyte plants such as *C. album* L. and *Atriplex canescens*. Spinach salt tolerance seems to be an interplay of high tissue tolerance to Na and Cl, salt-tolerance genes expressed in EBCs and EBC-free leaves, and the homeostasis of K. These genes, previously reported to be expressed in roots, have important roles in salinity tolerance such as salt sequestration into vacuoles, extrusion of salts from roots to soil, ion homeostasis under salinity and mineral deficiency, etc. These genes must be further studied under combined salinity stress and NPK deficiency to increase our knowledge of the complexity of salinity tolerance mechanisms. While further research is needed to better understand the function of spinach EBCs, recent evidence suggests that these structures may contribute to ion homeostasis or insect resistance.

Our current findings establish that spinach leaves accumulate Na and Cl at macronutrient levels, and higher levels than quinoa, without any visual toxicity symptoms; that spinach EBCs accumulate mostly K and Cl, and Na to a lesser extent, mainly under high salinity; that spinach EBCs overexpress genes that are involved in ion homeostasis. These findings, added to our previous reports that spinach maintained its N, P, and K homeostasis under both salinity stress and K deficiency and favored K over Na, all hallmarks of halophytic plants, suggest that spinach should be reclassified from a glycophyte to a facultative halophyte, as other members of the Amaranthaceae.

## Methods

### Comparison between spinach and quinoa genotypes at high salinity level in pot experiment

An experiment was carried out in a greenhouse at the United States Salinity Laboratory (USSL), Riverside, California, to compare salt accumulation in EBCs of two spinach (*S. oleracea* L.) cultivars (Gazelle and Seaside) with two quinoa (*C. quinoa* Willd.) genotypes (CPAC09 and CPAC11) developed by EMBRAPA Cerrados, Planaltina, Brazil. In March 2023, spinach and quinoa were planted in pots containing 9 kg of soil, with two plants per pot for quinoa, and three plants per pot for spinach. After 25 days of seed germination, the plants with 4–6 true leaves were irrigated with waters of EC_iw_ of 2 dS m^−1^ (control) and 25 dS m^−1^ (high-salinity). Both irrigation solutions were balanced with mixed salts to ensure adequate plant fertilization. The ionic compositions of the waters were adjusted based on the meq/L salt ratio present in seawater with a proportion of Cl^−^:Na^+^:Mg^2+^:SO_4_^2−^:Ca^2+^ = 25.5:22.7:5.2:1.3:1.0 (Supplementary Table 2). Both treatments provided the basic plant nutrition through a half-strength modified Hoagland’s nutrient solution with the potassium concentration fixed at 5 mmol_c_ L^−1^, based on previous results for K requirements of spinach under salinity^30^. The experiment was conducted with four replicates per treatment.

To determine Na and Cl concentration in spinach and quinoa leaves, the plants were harvested before flowering, a stage by which plants have accumulated most of the nutrients needed for growth and development, and after 52 days of saline-water application. Before the application of saline waters, pot soil was allowed to achieve field capacity, and a volume of water was calculated to apply a leaching fraction of 0.4 (40% more water than required by evapotranspiration) so the soil solution would have a continuous and homogeneous salt concentration around the root zone. This volume gradually increased as plants grew taller and increased their biomass.

Na and K were determined from nitric acid digestions of oven-dried and ground plant material by Inductively Coupled Plasma Optical Emission Spectrometry (ICP-OES, 3300DV, Perkin-Elmer Corp., Waltham, MA, USA), Cl was determined from nitric-acetic acid extracts by amperometric titration.

To determine whether EBCs are present across diverse spinach genotypes, we examined 11 additional cultivars: Cornell ID #148 (PI 608712), Cornell ID #87 (PI 173131), Dikenli (PI 171861), Monstrans Viroflag (PI 176371), Viking (NSL 28218), Polag Benaresi (PI 163309), Victoria (PI 179595), CGN9629 (PI 206753), Dikensiz (PI 169668), 11 (PI 103063), and Palek (PI 220668).

### Comparison of spinach and quinoa performances at different salinity levels in the lysimeter system

To compare the performance of spinach (ʻCGN 9629ʼ) and quinoa (ʻCPAC11ʼ) at different salinity levels, seeds were germinated in pots containing vermiculite. At the two-leaf stage, seedlings were transplanted into lysimeter sand tanks measuring 1.2 m × 0.6 m × 0.5 m. Each set of three tanks was connected to an 890 L irrigation reservoir located in the greenhouse basement. Irrigation water was delivered via polyvinyl pipes, and excess water was returned to the reservoirs by gravity for reuse. The system featured automated control of irrigation frequency and duration, and water consumption was routinely monitored.

The experiment was conducted in a randomized complete block design with three replications and four salinity treatments (EC_iw_ of 2, 8, 16, and 24 dS m^−^^1^) (Supplementary Table 3). For the first four weeks after transplanting, plants were irrigated once daily with the nutrient solution. Salinity treatments began at the six-leaf stage. Salinity was gradually increased to avoid osmotic shock: 8 dS m^−^^1^ on the first day, 16 dS m^−^^1^ on the second day, and 24 dS m^−^^1^ on the third day. Plants were grown under these conditions for three weeks before harvest. At harvest, plant height, shoot dry weight, and root dry weight were recorded. Roots and leaves were dried separately at 70 °C for 96 h before weighing.

### Scanning electron microscopy (SEM) of bladder cells

Sections of young leaves with EBC’s were fixed with formalin-acetic acid-alcohol, dehydrated in ethanol to 100%, critical-point dried, mounted on aluminum stubs, sputter-coated with Au/Pd and imaged at the University of Wisconsin, Stevens Point with an Hitachi S-3400 SEM in secondary electron mode at an accelerating voltage of 5 kV.

### Scanning electron microscopy with energy-dispersive X-ray spectrometry (SEM-EDS)

EBCs were removed from the youngest leaves of quinoa and spinach with a small metal spatula after 60 days of irrigation with waters of low- and high-salinity to evaluate the accumulation of salt ions using SEM-EDS. Spinach and quinoa bladders were carefully scraped onto the top of aluminum stubs without any tape to prevent contamination. The stubs were previously washed with molecular water (18.2 megohms) and air-dried to eliminate the water interference. The bladders were sputtered with a layer of gold. The SEM-EDS semi-quantitative analysis was carried out at the University of California in Riverside in a TESCAN Vega3 SBH microscope (TESCAN USA, Inc., Pleasanton, CA, USA). The energy of 15 kV was used on the bladders of both species to provide the best readings for all elements analyzed.

Semi-quantitative analysis of K, Cl, and Na concentrations of two cultivars each of quinoa (CPAC09 and CPAC11) and spinach (Gazelle and Seaside) was carried out using SEM-EDS. The values in g kg^−^^1^ of each element were calculated from the normalized element mass concentration (in %) provided by each element intensity in a spectrum signal generated by its energy obtained by SEM-EDS at 15 keV. Each value was originated from three different readings at three points in the gland and performed for three different EBCs (*n* = 9).

### Membrane staining and confocal microscopy

Tissue samples from each plant were stained with Nile Red to visualize cell membranes of epidermal cell structures^50^. Briefly, a stock solution of 1 mg mL^−^^1^ Nile Red or Nile Blue A Oxazone (Sigma-Aldrich Inc., St. Louis, MO, USA) prepared in 100% DMSO (Sigma-Aldrich Inc., St. Louis, MO, USA) was diluted to 1 μg mL^−^^1^ in sterile distilled water to create a working solution. Dissected leaf tissue samples were incubated in a working solution for 5–10 min and then rinsed with sterile water for 1 min before mounting.

EBCs were visualized using an inverted Zeiss LSM 900 (Zeiss, Dublin, CA, USA) confocal microscope equipped with an EC Plan-NeoFluar 10x/0.3 NA objective. Nile Red (excitation wavelength 561 nm) and chlorophyll fluorescence (excitation wavelength 640 nm) were observed simultaneously by collected emission spectra from 400 to 650 nm and 650 to 700 nm, respectively. Z-series images were collected in 1 μm intervals to image the entire epidermal structure and converted into maximum-intensity projections using the Zeiss Zen Blue Software.

### Light microscopy

Most samples were imaged with a Leica S9 dissecting microscope or Leica DM 750 compound microscope with a built-in cameras. The cameras were calibrated against measurement standards and scale bars merged with each image. Stacked in-focus images were made from a Z-series of images using Helicon Focus ver. 8.2.2 Pro software.

### Expression analysis

To compare gene expressions in bladders and other leaf cells, two spinach varieties (ʻGazelleʼ and ʻSeasideʼ) were used. The bladders were manually removed from the selected leaves using a spatula. RNA was extracted from both bladders and the remaining leaf tissues using the TRIzol^®^ solution (Invitrogen, Carlsbad, CA, USA) and was subsequently treated with DNase I (Thermo Scientific, Waltham, MA, USA), adhering to the guidelines provided by the manufacturers. The quantitative Reverse Transcription-PCR (qRT-PCR) procedures were conducted using the iTaq™ Universal SYBR® Green One-Step Kit on a Bio-Rad CFX96 thermal cycler (Bio-Rad Laboratories, Hercules, CA, USA).

A set of 17 genes involved in Na, Cl, and K transport, ion compartmentalization, and tissue tolerance were examined for the expression analyses. Each PCR reaction mixture, with a total volume of 10 µl, comprised 20 ng of RNA, 0.75 µM of specific primers (Supplementary Table 1), 0.125 µL of iScript™ Reverse Transcriptase, and 5 µL of the 2x SYBR® Green Reaction mix. These experiments were repeated across three biological and two technical replicates. The PCR protocol involved an initial phase at 50 °C for 10 min, a 1-min interval at 95 °C, followed by 40 cycles consisting of 10 s at 95 °C for denaturation, 30 s at 57 °C for annealing, and a 30 s extension at 68 °C. Relative expression levels were determined using the comparative 2^−ΔΔCT^ method^51^. For normalization of gene expression, reference genes used were spinach ACTIN (Spov3_chr2.02265), Actdf (Spov3_chr6.00169), and GAPDH (Sov3_C0001.00042).

Differences in gene expression were quantified by contrasting the cycle threshold values of the target gene against those of the reference genes. The formula employed for this calculation was:

Normalized expression _sample (GOI)_ = [RQ_sample (GOI)_]/[RQ_sample (ref 1__)_ x RQ_sample (ref 2)_ x….x RQ_sample (ref *n*)_]^1/*n*^

In the formula, RQ is the relative amount of a sample, ref is the reference target gene in a run that includes one or more reference targets in each sample, and GOI is the gene of interest.

### Statistics and Reproducibility

All analyses were performed in SAS Studio (SAS Institute Inc., Cary, NC, USA). Normality and homogeneity of variances were assessed with the Shapiro–Wilk and Levene tests, respectively; assumptions were considered met at *p* > 0.05. Data meeting assumptions were analyzed by two-way ANOVA (species × salinity; *α* = 0.05). Tukey’s HSD was used for post hoc comparisons: (i) among salinity levels within each species and (ii) between species at each salinity (*α* = 0.05).

To assess salinity effects in spinach and quinoa, we performed one-way ANOVA within each species across salinity treatments (*α* = 0.05) for plant height, shoot dry weight, and root dry weight. When significant, Tukey’s HSD tested pairwise differences among salinity levels within that species (*α* = 0.05). Sample size was *n* = 9 biologically independent plants per group.

Gene-expression differences between EBCs and EBC-free leaves were tested with two-tailed t-tests. For each condition, *n* = 3 biologically independent plants were analyzed; two technical qPCR replicates per biological sample were averaged. Significance is indicated as *P* < 0.05 (*) and *P* < 0.01 (**). For boxplots, horizontal line indicates the median, boxes the interquartile range, “*x*” the mean, whiskers 1.5 × IQR, and points individual biological replicates.

### Reporting summary

Further information on research design is available in the Nature Portfolio Reporting Summary linked to this article.

## Supplementary information

Supplementary Information

Description of Additional Supplementary Files

Supplementary Data 1

Supplementary Data 2

Supplementary Data 3

Supplementary Data 4

Supplementary Data 5

Reporting Summary

## Data Availability

Raw data are provided as follows: Table 1—Supplementary Data 1; Fig. 6—Supplementary Data 2; Table 2—Supplementary Data 3; Table 3—Supplementary Data 4; Fig. 7 and Supplementary Fig. 1—Supplementary Data 5. All other data are available from the corresponding author upon reasonable request.
