# Supplementary material for: Spinach (Spinacia oleracea) has epidermal bladder cells and exhibits characteristics of a facultative halophyte
Source: Commun Biol. 2025 Nov 29;8:1806. doi: 10.1038/s42003-025-08936-6 (PMC12727796; doi:10.1038/s42003-025-08936-6)
Supplement: Supplementary file 1 — Supplementary Information [file 42003_2025_8936_MOESM1_ESM.pdf]

Supplementary Table 1. Primers used for expression analysis

| Gene Name | Primer Name | Primer Sequence (5' - 3') |
|---|---|---|
| *NHX1* | Spo20154_1F | GCACATTGCAGGTACTTAATCAG |
| | Spo20154_1R | CGAAGCTCTGGATAGCGTTAAA |
| *NHX2* | Spo09537_1F | CCCTTCTTCTTGGCATAGTGT |
| | Spo09537_1R | CTTAACCTGAAACCCAGCATTG |
| *SOS1* | Spo00867_1F | ACGGTATCCGCATTTGGG |
| | Spo00867_1R | CTGCACACCTCTTTATCTGGT |
| *SOS2* | Spo15557_1F | ATGGTTGAGCAGATTAGACGT |
| | Spo15557_1R | TCGGCTGGCTAAAACCTC |
| *SOS3* | Spo02501_1F | GCCTCAGAAACACCATTCAC |
| | Spo02501_1R | GTTGGAACTCTTCCCTGTGA |
| *HKT1* | Spo18318_2F | CATCACTGAGAGCACCAGTTTA |
| | Spo18318_2R | TCCCATATGCACTTACGACTTC |
| *AKT1* | Spo12230_2F | GCTTTTCCAGCTGGTTTCAG |
| | Spo12230_2R | GGATGATAAATTCCATTGCACCAG |
| *NPF2.4* | Spo16635_1F | CTCCTTACTGGGCGCAATAAT |
| | Spo16635_1R | CAAGGCTTGATGGAGGGTTAT |
| *SLAH1* | Spo20847_1F | TTCATGTCCTTGGTAAGTAGACC |
| | Spo20847_1R | TGCTAGTGCTAGTATTGTCATAGG |
| *CCC* | Spo03495_1F | TGCAATGAAGGGTGGTGG |
| | Spo03495_1R | AACACATAGAGAGATGCAGCA |
| *ALMT12* | Spo02965_2F | GACGAGGCCATGGCATTATAT |
| | Spo02965_2R | GACGAAGAACTGCTCCTACTTT |
| *CLCc* | Spo04588_2F | GCATCTCCATACACTGTGCT |
| | Spo04588_2R | AATTGGAGATCTCCCTTGACTC |
| *CLCg* | Spo08512_1F | AATGGCTTCTTGGTGGAGAA |
| | Spo08512_1R | CTTGCCACTGTAGCACAAATC |
| *Spo09736 (EXLB1)* | Spo09736_1F | TCCAACTGGAGCATGTGG |
| | Spo09736_1R | CCGTACCTGATAGCAACCAC |
| *Spo11258 (E3-ubiquitin ligase)* | Spo11258_1F | CCCTTAACTCATGCTCCTCAC |
| | Spo11258_1R | CGACGTGATCAATGGAGATGATA |
| *Spo15968 (SLAH2-like)* | Spo15968_1F | GCAGATAACAGAGAAACTGAAGTAAC |
| | Spo15968_1R | CATGTGGAACAACTTTGGATGAA |
| *Spo19814 (EGY3)* | Spo19814_1F | AACAAGTTGAAGAATGGCTTTGG |
| | Spo19814_1R | GGCTTCCTCAAATTCCCTATGA |
| *GAPDH* | Spo21203_2F | GTGTCAACGAGGAAGGTTACA |
| | Spo21203_2R | CCCTTGATGATGCCAAATTTCT |
| *ACTIN* | Spo18993_2F | GGTCGTACTACTGGTATTGTATTGG |
| | Spo18993_2R | AGATCACGTCCAGCCAAATC |
| *Actdf* | Spo17116_1F | CAAAAGGTCAAACTCTCTTTCTGG |
| | Spo17116_1R | GCGATGATCCTTCTTCCTCTTTA |

Supplementary Table 2. Description and ionic composition of the saline waters used for the pot experiment.

| Treatment | $EC_{iw}$ (dS $m^{-1}$) | Ion concentration ($mmol_c$ $L^{-1}$) | | | | | | | | |
|---|---|---|---|---|---|---|---|---|---|---|
| | | $Cl^-$ | $SO_4^{2-}$ | $NO_3^-$ | $PO_4^{3-}$ | $HCO_3^-$ | $Na^+$ | $K^+$ | $Ca^{2+}$ | $Mg^{2+}$ |
| T0 | 2 | 6.52 | 2.02 | 5.39 | 1.5 | 3.07 | 6.69 | 6.58 | 3.2 | 2.05 |
| T1 | 8 | 61.52 | 14.52 | 5.39 | 1.5 | 3.07 | 51.69 | 6.58 | 15.7 | 12.05 |
| T2 | 16 | 137.02 | 29.02 | 5.39 | 1.5 | 3.07 | 113.69 | 6.58 | 31.2 | 24.55 |
| T3 | 24 | 206.52 | 43.02 | 5.39 | 1.5 | 3.07 | 171.69 | 6.58 | 45.7 | 36.05 |

Supplementary Table 3. Description and ionic composition of the saline waters used for the lysimeter experiment

| Target $EC_{iw}$ | Calculated $EC_{iw}$ | Ion concentration ($mmol_c$ $L^{-1}$) | | | | | | | |
|---|---|---|---|---|---|---|---|---|---|
| (dS $m^{-1}$) | (dS $m^{-1}$) | $Na^+$ | $Ca^{2+}$ | $Mg^{2+}$ | $SO_4^{2-}$ | $Cl^-$ | $K^+$ | $NO_3^-$ | $PO_4^{3-}$ |
| 2 | 1.89 | 2.1 | 1.7 | 3.3 | 0.6 | 3.7 | 4.9 | 7.61 | 1.5 |
| 25 | 24.8 | 196.1 | 5.4 | 44.2 | 21.5 | 221.4 | 4.9 | 7.61 | 1.5 |

**Supplementary Figure 1. Gene expression profiles of salt tolerance–related genes in epidermal bladder cells (EBCs) and EBC-free leaf tissues of spinach cultivars 'Gazelle' and 'Seaside' irrigated with 25 dS m$^{-1}$ saline water.**

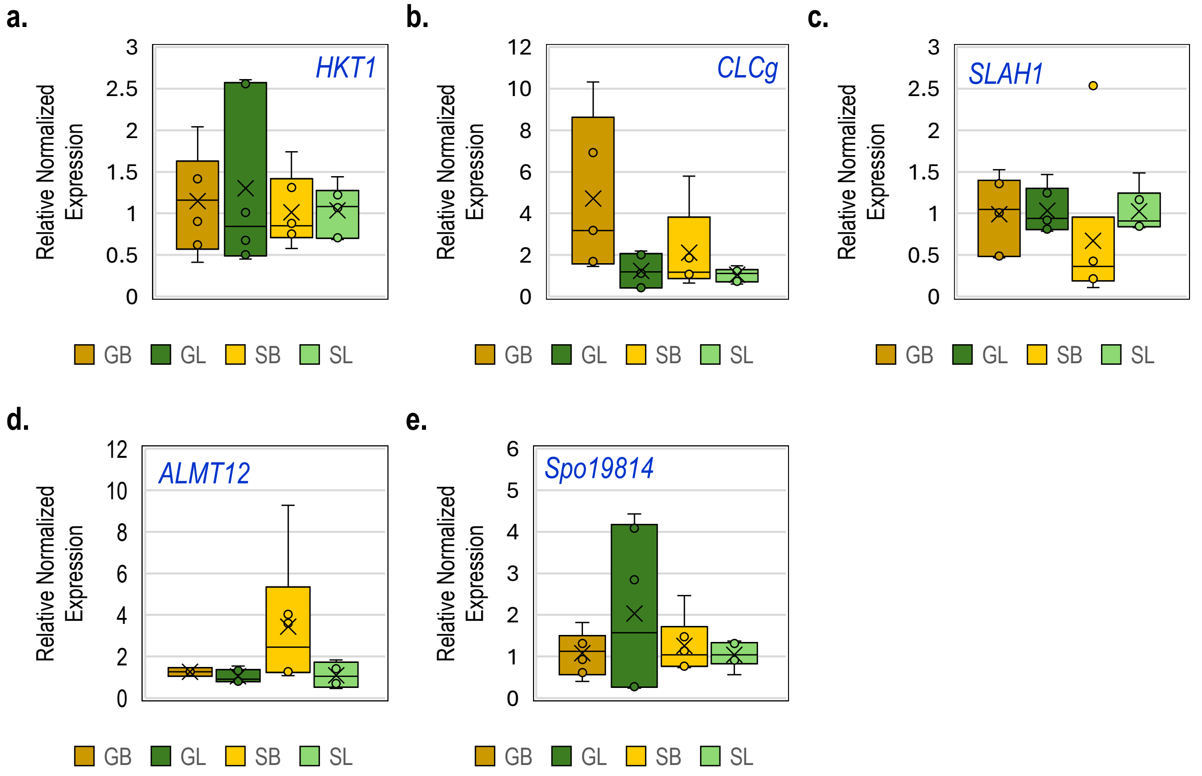


**a** *HKT1*. **b** *CLCc*. **c** *SLAH1*. **d** *ALMT12*. **e** *Spo19814*. GB, 'Gazelle' EBC; GL, 'Gazelle' leaves devoid of EBCs; SB, 'Seaside' EBC; SL, 'Seaside' leaves devoid of EBCs;
