# Supplementary material for: Spinach (Spinacia oleracea) has epidermal bladder cells and exhibits characteristics of a facultative halophyte
Source: Commun Biol. 2025 Nov 29;8:1806. doi: 10.1038/s42003-025-08936-6 (PMC12727796; doi:10.1038/s42003-025-08936-6)
Supplement: Supplementary file 2 — Description of Additional Supplementary Files [file 42003_2025_8936_MOESM2_ESM.pdf]

Description of Additional Supplementary Files

File name: Supplementary data 1

Description: Ion concentration (K, Cl, and Na) in quinoa and spinach EBCs

File name: Supplementary data 2

Description: Plant height, shoot dry weight, root dry weight of spinach and quinoa under four salinity treatments.

File name: Supplementary data 3

Description: Ion concentration (K, Cl, and Na) in quinoa and spinach leaves

File name: Supplementary data 4

Description: Ion concentration (K, Cl, and Na) in quinoa and spinach roots

File name: Supplementary data 5

Description: Expression data for various salt tolerance-related genes in epidermal bladder cells (EBCs) and EBC-free leaf tissues of spinach cultivars ‘Gazelle’ and ‘Seaside’ irrigated with 25 dS m^{-1} saline water.
